# **Personalized medicine and allergen immunotherapy: the beginning of a new era?**

**DOI:** 10.1186/s12948-021-00150-z

**Published:** 2021-07-07

**Authors:** Cristoforo Incorvaia, Erminia Ridolo, Diego Bagnasco, Silvia Scurati, Giorgio Walter Canonica

**Affiliations:** 1 Cardiac/Pulmonary Rehabilitation, ASST Pini-CTO, Milan, Italy; 2 Allergy and Clinical Immunology, Medicine and Surgery Department, University of Parma, Parma, Italy; 3 Allergy and Respiratory Diseases, DIMI Department of Internal Medicine, University of Genoa, Ospedale Policlinico San Martino, Genoa, Italy; 4 Stallergenes Greer Medical Affairs Department, Antony, France; 5 Personalized Medicine, Asthma & Allergy-Humanitas Clinical and Research Center, IRCCS, Rozzano, MI Italy

**Keywords:** Allergen immunotherapy, Personalized medicine, Asthma, Allergic rhinosinusitis

## Abstract

The concept of personalized medicine as a diagnostic and therapeutic approach tailored to the medical needs of each patient is currently revolutionizing all fields of medicine and in particular allergology. Allergen immunotherapy (AIT) meets the three main needs for precision medicine: identification of molecular mechanism of disease, diagnostic tools for the mechanism and treatment blocking the mechanism itself. AIT adapts to the spectrum of specific IgE of each individual subject, changing the course and natural history of the disease, so is a clear model of precision and personalized medicine. This first step before the prescription of AIT is to define the sensitization profile of the patient; after that, the healthcare professional has numerous levers for adapting the treatment to the physio-pathological mechanisms involved. AIT allows to adapt treatments to the profile of the patients, but also to the its preferences, to ensure optimal treatment efficacy, resulting in an agile and personalized approach, with the aim to ensure adherence to the treatment, which is usually quite low. AIT also broadens the field of possibilities for healthcare professionals and patients, by allowing to choose the galenic formulation according to patient preferences and on the basis of their clinical history, adapting the product composition to the patient’s sensitization profiles and the underlying biological mechanisms identified at the diagnostic stage, while guaranteeing quality of the prescribed product as the production of allergens and allergoids is today more regulated than in the past years. In the management of AIT, it is also possible to involve patients in decisions throughout their care pathway thanks to multiple services, offering personalized follow-up and support, to ensure the highest treatment efficacy levels, and recalling medication intake, medical appointments and prescription renewals.

## Background

Starting from its distant introduction as an empirical treatment of hay fever, allergen immunotherapy (AIT) has been developed to achieve recognition of scientific evidence of efficacy and safety [1]. The progress has mainly concerned the qualitative improvement of allergen extracts for AIT, which have recently obtained pharmaceutical quality recognition from regulatory agencies [2]. However, the efficacy of a product for AIT demonstrated in controlled trials may not be reproduced in current practice if the prescription is not targeted on the individual characteristics of the patient, thus influencing negative opinions on such treatment.

The concept of personalized medicine as a diagnostic and therapeutic approach tailored to the medical needs of each patient [3] is currently revolutionizing all fields of medicine and in particular allergology. New opportunities are arising: technological progress combined with current scientific advances suggest the possibility of developing new comprehensive approaches to better manage patient health and target therapies to achieve the best outcomes in the management of a patient’s allergy. Actually, AIT meets the three main needs for precision medicine, which are identification of molecular mechanism of disease, diagnostic tools for the molecular mechanism and treatment blocking the mechanism [4].

To cope with the multiplication of allergic conditions, which are increasingly complex and multifactorial, the shift towards this new medicine has become inevitable. Allergology is therefore at a major turning point in its history: AIT and recent medical innovations are paving the way for a realistic “tailor-made approach” to the treatment and care of patients with respiratory allergies.

Indeed, such issue faces many challenges related to the patient’s management. Today, the increase in respiratory allergy triggers, mainly related to air pollution and climate change is accompanied by an increase in the prevalence of asthma, rhinitis and rhino-conjunctivitis [5]. In addition, respiratory allergies are still poorly diagnosed and their symptoms insufficiently controlled, thus illustrating the current limitations of care pathways for the management of allergy. They therefore generate high, sometimes avoidable, costs for the healthcare system and their societal impact is even more significant as they impair patient quality of life and are associated with severe comorbidities.

These challenges act as key drivers of the shift towards the tailoring of medical treatments to the individual characteristics, needs and preferences of a patient during all stages of care, including prevention, diagnosis, treatment and follow-up. This personalized medicine model is based on four fundamental/rooted pillars: personalization, prediction, prevention and patient participation.

The evolutionary path of the AIT approach has historically started from a prescription based on the etiological factor related to the mechanisms of the disease, proceeding through a better knowledge of the endotype, what today is defined as precision medicine, to a personalized medicine. The concept of personalized medicine comes directly by the one of personalized medicine, but enriches it with careful analysis and observation of the peculiar features of the patient we are treating. Just AIT, because it adapts to the spectrum of specific IgE of each individual subject, changing the course and natural history of the disease, is a clear model of precision and personalized medicine [6].

The prescription of AIT must first be based on a personalized diagnosis of the clinical phenotype using, for example, biomarkers that can guide medical prognosis and the targeting of treatments.

Regarding the possible predictive biomarkers, several have been hypothesized and analysed, although there are no predictive ones. The EAACI Task Force "Biomarkers for monitoring the clinical efficacy of allergen Immunotherapy" has acted with the aim to analyse possible predictive biomarkers of efficacy, indicating as main markers the high levels of specific serum IgE and the symptoms in contact with the specific allergen. Furthermore, the ratio of specific IgE vs total IgE (sIgE/tIgE) was also evaluated, particularly in AIT household dust mite (HDM) or grass pollen, with controversial results, where some studies indicated the ratio as a possible response marker, and others failed to detect a real reliability. Several studies indicated a possible correlation between several subtypes of IgG (IgG1, sIgG4) and clinical outcomes, but another time without general consensus [7].

This first step must be based on a precise characterization of the sensitization profile using tools adapted to the patient profile ("classic" in-vivo IgE antibody tests and/or in-vitro molecular allergy tests). This diagnosis will then guide the definition of therapeutic objectives tailored to each patient. On the one hand, AIT can be used as an effective and personalized etiological treatment. Indeed, the healthcare professional will have numerous levers for adapting the treatment to the physio-pathological mechanisms involved (allergen composition, schedule and dosage of administration and efficacy measures, and definition of monitoring modalities). On the other hand, AIT is also prescribed to develop the patient's immunological memory so as to for example, prevent the onset of asthma, new sensitizations and reduce asthma medication intake. Importantly, patient empowerment during all stages of care, through the definition of personalized treatment plans and shared medical decision-making, is necessary to fully involve individuals with respiratory allergies in decisions about their care. As already said before AIT is a paradigmatic therapeutic approach to precision and personalized medicine, and in this field molecular diagnostics can provide important information about the patient and his therapy. For several years now, not only has the use of molecular diagnostics been advocated, but protocols have also been created that indicate how to use this method in order to identify the best approach of AIT. It has been observed that, after reanalysing patients with molecular diagnostics, the choice of AIT previously prescribed could be reviewed in almost half of the patients. For these reasons, to make the therapy with AIT more and more effective, the molecular diagnosis, for the patients to be treated, must be encouraged [8].

Finally, driven by the challenges mentioned above and nourished and disseminated by this first model of personalized medicine, future developments in allergology will favour the growth and deployment of a new model of "customized" medicine. The integration of large amounts of omics datasets, the discovery of new biomarkers of endotypes, treatment responses and monitoring, and the development of targeted biological therapies will make it possible to stop the initiation or halt the progression of the allergic march, to reduce the burden of disease and to increase patient satisfaction.

## Personalizing allergen immunotherapy treatments: the field of possibilities

The current context is a real breeding ground for the development of personalized medicine in allergology. In particular, the possible modulation of AIT treatments allows for the broadening of the allergist's field of action. The allergist has a multitude of options to adapt treatment and follow-up to the individual characteristics of each patient. Today, the emergence of new behaviours and profound changes in the environment are the new scientific evidence of the need to reshape the management of patients with respiratory allergies: first, patients adopt new behaviours. Some patients, called sentinel patients, have learned to identify with the utmost acuity the slightest warning signs of a seizure. They have developed a sensory perception of early symptoms that allows them to process individually the available information. It results in a “personal” semiology on which they base a diagnosis, allowing them not only to cope with crises but also to better manage, daily, their disease. Throughout their semiology—which is the result of a long and progressive self-learning process—perception and reasoning complement each other. Nevertheless, patients are still not able to notice in time the variation of their symptoms, and less suitable for modular therapy. In general, the problem of adherence to treatment is always present, which is statistically quite low among allergic/asthma patients.

Secondly, global warming causes significant changes in patient external environments. This has the following consequences:

- A prolonged pollination period, due to higher temperatures and air humidity;
- An increase in the amount of pollen in the air, due to faster and more extensive plant growth;
- Changes in the weather, such as thunderstorms during pollen seasons, may induce hydration of pollen grains and their fragmentation, generating atmospheric biological aerosols that carry allergens;
- Changes in the distribution areas of trees and herbaceous plants that spread over territories whose previous climatic conditions were not favourable to their development.
- An increase of environmental pollution, lengthy described as triggering factor for poor control of allergic symptoms [9].

## Conclusions

In light of these developments, it is necessary to adapt treatments to the profile but also to the preferences of each patient, to ensure optimal treatment efficacy. AIT allows this agile and personalized approach. The healthcare professional will then have to build multiple efficacy measures adapted to the therapeutic objectives previously defined during the diagnosis. AIT broadens the field of possibilities for healthcare professionals and patients by allowing to:

- Choose the galenic formulation according to patient preferences and on the basis of their clinical history. For example, the liquid form of sublingual AIT (SLIT) will be more suitable for children or particularly sensitive patients requiring dose and schedule adaptation while the tablets will be appropriate, for a smartest usability, for example in active and traveling patients.
- Adapt product composition to patient sensitization profiles and the underlying biological mechanisms identified at the diagnostic stage.
  With the important step forward, done in AIT extracts quality, now we can be better confident about the quality of the prescribed product. In recent years, in fact, the attention of regulatory centres and companies has increased, so that the production of allergens and allergoids is well regulated, with the aim of marketing only quality products. The premise is to choose validated product, i.e. products with documented scientific evidence of efficacy and safety.
  Different allergenic extracts may be prescribed, depending on patient sensitization profiles (single extracts for mono-allergic patients or poly-allergic patients with one clearly most bothersome causal allergen)—as well as mixtures for poly-allergic or poly-medicate patients, or in one with a tendency to forget treatment intake and a high likelihood of non-adherence.
- Chose the favourite administration route, with a shared choice between physicians and patients, between SCIT, SLIT drops and SLIT tablets, patient could be better motivated in a correct management and adherence to the therapy prescribed
- Adapt the dosage and schedule of administration of the treatment, during initiation and maintenance protocols, according to the maximum tolerated dose, patient susceptibility, exposure to allergens, intercurrent events and/or the occurrence of side effects, concomitant pathologies or allergies, the observed level of treatment efficacy or the patient's behaviour;
- Define patient-specific treatment goals based on patient clinical histories and expressed preferences, e.g.:
  - Prevent the onset of concomitant asthma and/or disease progression in children with particular genetic susceptibility;
  - Control of allergy symptoms;
  - Reduce inhaled corticosteroid (ICS) intake by acting directly on the aetiology of allergy symptoms.

In addition to the management of AIT, it is also possible to involve patients in decisions throughout their care pathway thanks to multiple services. Personalized follow-up and support will ensure the highest treatment efficacy levels. Among the wide variety of support services available, it is for example possible to:

- Use mobile applications and share information about the allergy and available treatments with the patient. Available applications allow, for example to:
  - Guarantee the transparency of the prescription and product circuit and enables the tracking of product delivery to the patient’s home.
  - Track pollen counts using patient locations;
  - Follow disease progression;
  - Monitor patient adherence to treatment;
  - Recall medication intake, medical appointments and prescription renewals.

**Fig. 1:**
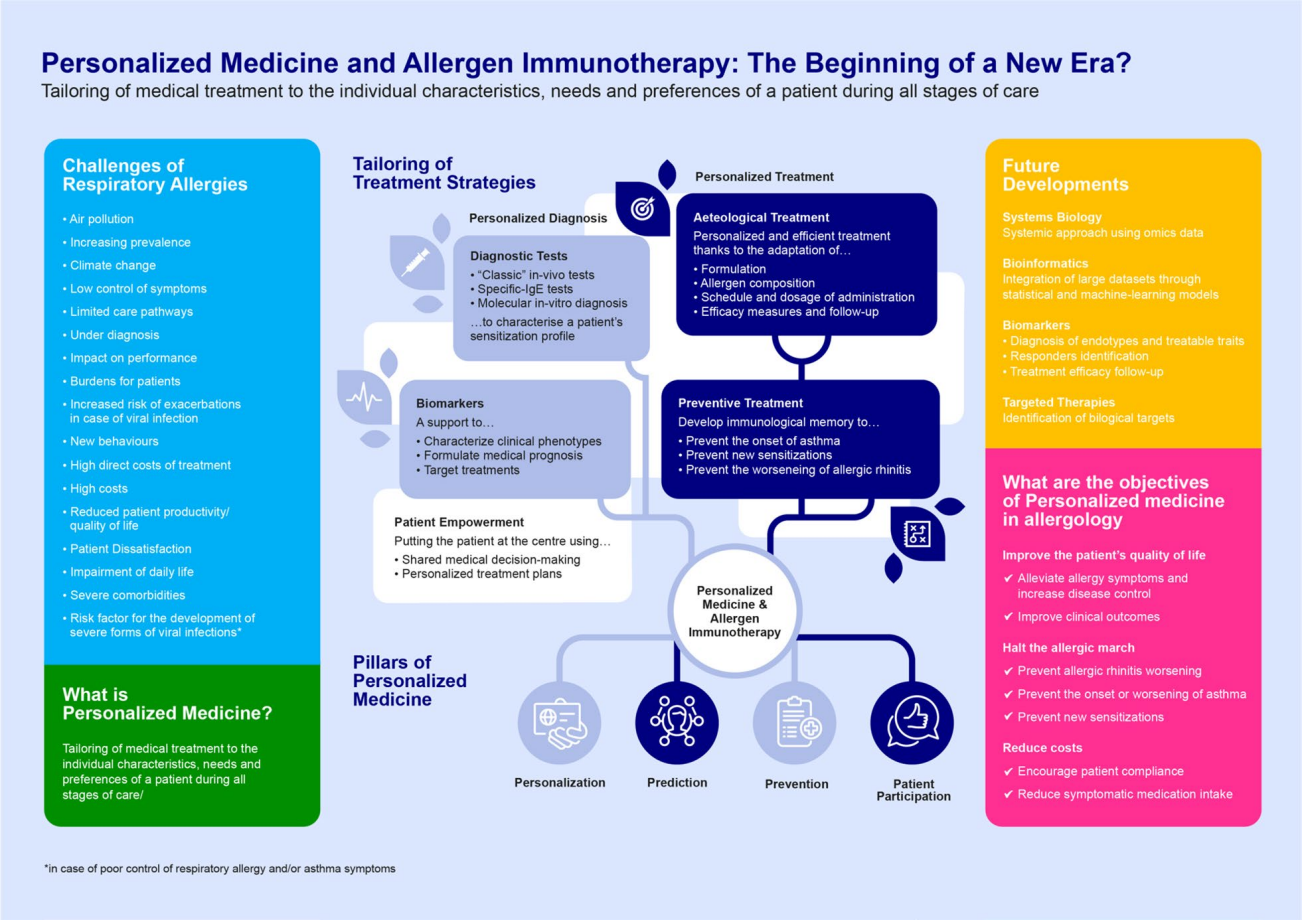

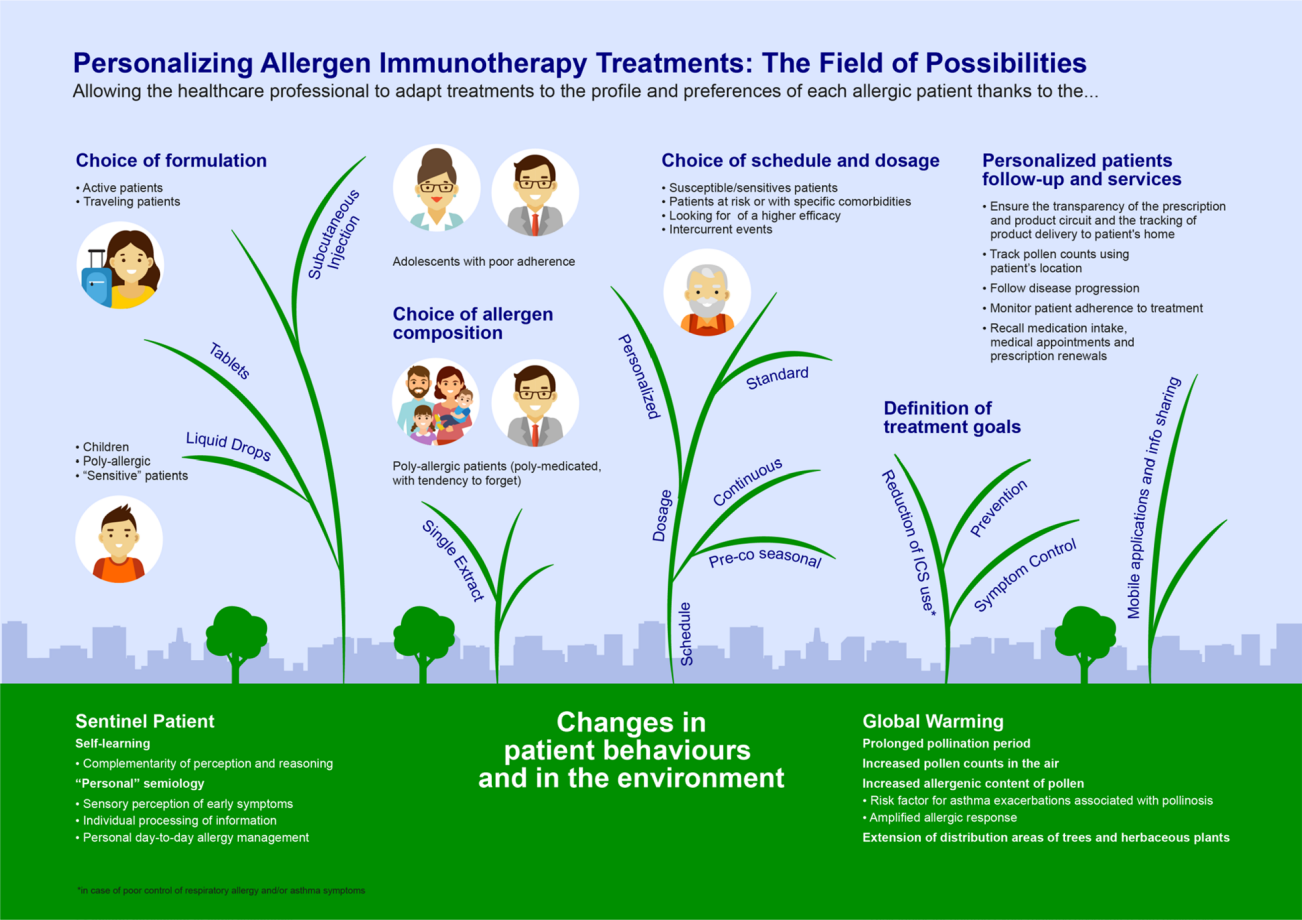
Essential features of allergen immunotherapy

Figure 1 summarizes the tailoring and the field of possibilities of personalizing AIT, which makes it feasible to develop therapeutic objectives and protocols adapted to each allergic patient, increasing treatment efficacy. This tailored approach enhances the expertise and role of the healthcare professional in developing, in collaboration with the patient, a personalized treatment plan [10].

## Data Availability

Not applicable.

## Abbreviations

AIT: Allergen immunotherapy
EAACI: European Academy of Allergy and Clinical Immunology
ICS: Inhaled corticosteroid

## References

[1] Passalacqua G, Canonica GW (2016). Allergen Immunotherapy: history and future developments. Immunol Allergy Clin N. Am.

[2] Bonertz A, Roberts GC, Hoefnagel M (2018). Challenges in the implementation of EAACI guidelines on allergen immunotherapy: a global perspective on the regulation of allergen products. Allergy.

[3] Hamburg MA, Collins FS (2010). The path to personalized medicine. N Engl J Med.

[4] Canonica GW, Bachert C, Hellings P, Ryan D, Valovirta E, Wickman M (2015). Allergen Immunotherapy (AIT): a prototype of Precision Medicine. World Allergy Organ J.

[5] D'Amato G, Chong-Neto HJ, Monge Ortega OP, Vitale C, Ansotegui IJ, Rosario N (2020). The effects of climate change on respiratory allergy and asthma induced by pollen and mold allergens. Allergy.

[6] Passalaqua G, Canonica GW (2015). AIT (allergen immunotherapy): a model for the "precision medicine". Clin Mol Allergy..

[7] Shamji MH, Kappen JH, Akdis M, Jensen-Jarolim E, Knol EF, Kleine-Tebbe J (2017). Biomarkers for monitoring clinical efficacy of allergen immunotherapy for allergic rhinoconjunctivitis and allergic asthma: an EAACI Position Paper. Allergy.

[8] Melioli G, Savi E, Crivellaro MA, Passalaqua G (2016). Potential of molecular based diagnostics and its impact on allergen immunotherapy. Asthma Res Pract..

[9] Naclerio R, Ansotegui IJ, Bousquet J, Canonica GW, D’Amato G, Rosario N (2020). International expert consensus on the management of allergic rhinitis (AR) aggravated by air pollutants: impact of air pollution on patients with AR: current knowledge and future strategies. World Allergy Organ J.

[10] Incorvaia C, Al-Ahmad M, Ansotegui IJ, Arasi S, Bachert C, Bos C (2021). Personalized medicine for allergy treatment: allergen immunotherapy still a unique and unmatched model. Allergy.

